# Switch to ocrelizumab in MS patients treated with natalizumab in extended interval dosing at high risk of PML: A 96-week follow-up pilot study

**DOI:** 10.3389/fimmu.2023.1086028

**Published:** 2023-02-01

**Authors:** Pilar Santiago-Setien, Cristina Barquín-Rego, Paula Hernández-Martínez, María Ezquerra-Marigomez, Marta Torres-Barquin, Cristina Menéndez-Garcia, Fernando Uriarte, Yésica Jiménez-López, Mercedes Misiego, Jose Ramón Sánchez de la Torre, Sonia Setien, Manuel Delgado-Alvarado, Javier Riancho

**Affiliations:** ^1^ Service of Neurology, Hospital Sierrallana-Institute of Research Valdecilla (IDIVAL), Torrelavega, Spain; ^2^ Service of Internal Medicine, Hospital Sierrallana-IDIVAL, Torrelavega, Spain; ^3^ Service of Radiology, Hospital Sierrallana, Torrelavega, Spain; ^4^ Service of Pharmacy, Hospital Sierrallana, Torrelavega, Spain; ^5^ Centro de Investigación en Red de Enfermedades Neurodegenerativas, CIBERNED, Instituto Carlos III, Madrid, Spain; ^6^ Department of Medicine and Psychiatry, University of Cantabria, Santander, Spain; ^7^ Red Española de Esclerosis Múltiple, Madrid, Spain

**Keywords:** extended interval dosing, natalizumab, ocrelizumab, multiple sclerosis, PML - progressive multifocal leukoencephalopathy

## Abstract

We aimed to assess the long-term safety and effectiveness of ocrelizumab in a cohort of patients with multiple sclerosis (MS) at high risk of progressive multifocal leukoencephalopathy (PML), previously treated with natalizumab in extending interval dosing (EID), who switched to ocrelizumab and to compare them with patients who continued EID-natalizumab. Thirty MS patients previously treated with natalizumab in EID (every 8 weeks) were included in this observational retrospective cohort study. Among them, 17 patients were switched to ocrelizumab and 13 continued with EID-natalizumab. Except for the John Cunningham virus (JCV) index, no significant differences were detected between both groups. Main outcome measures included: annualized relapse rate (ARR), radiological activity, disability progression, and the NEDA-3 index. Patients were followed for 96 weeks. The median washout period in ocrelizumab-switchers was 6 weeks. Among them, AAR and radiological activity during follow-up were 0.03, without significant differences in comparison with the previous period on natalizumab-EID. The comparison between ocrelizumab-switchers and patients continuing on EID-natalizumab showed no significant differences in AAR, radiological activity, or disability progression. However, the proportion of patients maintaining a NEDA-3 status in week 96 was slightly superior among ocrelizumab-switchers (94 vs 69%). No serious adverse events were observed in any group. In conclusion, switching from EID-natalizumab to ocrelizumab can be considered as a therapeutic option, particularly in patients with MS at high risk of PML, to mitigate the risks of both PML and disease reactivation.

## Introduction

The humanized monoclonal antibody natalizumab (Tysabri^®^; Biogen-Idec, Cambridge, MA, USA) is directed against the α4 subunit of α4β1 and α4β7 integrins preventing the entry of the circulating mononuclear cells into the central nervous system (CNS) through the blood-brain barrier. Natalizumab is one of the most effective and rapidly acting therapies for the treatment of relapsing-remitting multiple sclerosis (rrMS) (1). Although natalizumab is usually well tolerated by the vast majority of patients, it has been associated with an increased risk of progressive multifocal leukoencephalopathy (PML), a rare life-threatening infection caused by the John Cunningham virus (JCV) (2, 3). Of note, PML concerns are the most frequent cause of natalizumab discontinuation (4). PML risk is remarkably high in patients previously treated with immunosuppressant drugs, in patients treated with natalizumab for more than 24 months, and in those positive for JCV antibodies (particularly in patients with an JCV index > 1.5) (3, 5). On the other hand, discontinuation of natalizumab has been associated with MS reactivation and rebound (5). Thus, the therapeutic management in fully responsive MS patients treated with natalizumab at high risk of PLM represents a challenging decision. In this line, the extension of the interval dosing (EID) from 4 to 5-8 weeks or the switch to other high efficacy therapies constitute the most frequent strategies. Recently, the NOVA study reported that the extending natalizumab administration to 6 weeks was related to a slight loss of effectiveness, assessed by radiological activity (6). Previously, several descriptive studies reporting a favorable profile of natalizumab in terms of both efficacy and safety had been published (7–11). Among them, we reported a series of 39 patients in whom natalizumab in EID following a standard administration regimen maintained its disease-modifying activity, and was safe and well tolerated for over 7 years (12). Regarding switching, the change from natalizumab to anti-CD20 therapies, and particularly, to ocrelizumab appears as one of the preferable options (13–17)

Ocrelizumab (Ocrevus ^®^), considered as a highly effective therapy, is a humanized anti-CD20 antibody approved for the treatment of both rrMS and primary progressive MS (18). However, the experience of switching from natalizumab in EID to ocrelizumab is very limited. To date, very few studies reporting patients’ course after natalizumab cessation have been published (15, 19).

In this study, we aimed to assess the safety and effectiveness of ocrelizumab in a cohort of patients with MS at high risk of PML previously treated with natalizumab in EID, as well as to compare them with another cohort who continued therapy with natalizumab in extended dose.

## Patients and methods

We conducted an observational retrospective cohort study with analysis of data collected during routine clinical practice (clinical and neuroimaging evaluation with cranial MRI every 3 and 6 months, respectively) at the MS clinic, in the Hospital Sierrallana, in Cantabria, Spain. The protocol was approved by the institutional review board (Comité de Ética de la Investigación con medicamentos de Cantabria [CEIm Cantabria], reference number: 2019.328) and the study was performed in accordance with the relevant guidelines and regulations.

The Inclusion criteria were as follows: i) a diagnosis of clinically definite relapsing-MS, according to the McDonald revised criteria (20); ii) age over 18 years; iii) history of treatment with SID of natalizumab (every 4 weeks) for at least 24 months that was then extended to EID (every 8 weeks); and iv) switch to ocrelizumab during 2019 due to high risk of PML (cohort 1) or current continuation with natalizumab in EID (cohort 2).

Clinical charts were reviewed to collect the following variables: sex, age at diagnosis, duration of treatment with natalizumab in SID, reason for natalizumab extension, duration of treatment with natalizumab in EID, reason for switching to ocrelizumab, washout period, clinical relapses, the Expanded Disability Status Scale (EDSS) score and magnetic resonance imaging (MRI) (lesion load, presence of gadolinium-enhanced lesions). Regarding the MRI follow-up, ocrelizumab switchers underwent brain 1.5T MRI at the end of the washout period, and then at month 6, 12, 18 and 24 after ocrelizumab initiation to exclude a carry-over PML, and to detect MS reactivations. Among natalizumab stayers, MRI was performed at month 6, 12, 18 and 24. In addition, we carefully checked for potential natalizumab-related adverse reactions, specifically PML.

The main outcome measures were as follows: i) the annualized relapse rate (ARR), ii) presence of brain MRI activity (considered as ≥2 new T2-hyperintense lesions and/or new gadolinium-enhancing lesions), iii) EDSS score, and iv) disability progression defined as an increase of 1.5, 1, or 0.5 points in the EDSS in patients with a previous score of 0, < 5.5, and ≥ 5.5, respectively. As an outcome parameter of global disease control, we estimated the no evidence of disease activity (NEDA-3) status, which includes the combined absence of clinical relapses, radiological activity, and disability progression.

Included patients were divided in two groups: i) patients continuing treatment with natalizumab in EID and, ii) patients switching to ocrelizumab.

Baseline characteristics were compared by the non-parametric Mann–Whitney U test and the Fisher exact test. Global differences in ARR and EDSS across groups were tested by the Mann-Whitney U test. Kaplan-Meier analyses were used to assess the proportion of patients who maintained their NEDA-3. Differences were then tested by the Gehan-Breslow-Wilcoxon test. Prism software (GraphPad Software Inc., San Diego, California) was used for statistical analysis.

## Results

Thirty patients (17 switchers to ocrelizumab and 13 non-switchers) were included in the study. All patients included in the study had been initially treated with natalizumab in standard regimen for at least 24 months and subsequently changed to an extended regimen due to safety reasons. Among non-switchers, one patient died during the follow-up period due to an unexpected event not related to the disease nor the treatment. Main patient characteristics are summarized in **Table 1**. The primary reason for switching to ocrelizumab was the concern of a high risk of PML. Excepting the JCV index, no significant differences in terms of gender, age of onset, inflammatory activity, disability, or number of natalizumab infusions were found between switchers and EID stayers. (**Table 1**).

**Table 1:** Main patients characteristics.

	Ocrelizumab (n=17)	Natalizumab (n=13)	p
Female	14 (82%)	10 (77%)	0.53
Age at MS onset	31.4 (8.09)	35 (7,71)	0.19
Prior use of DMTs	11 (11IFN)	9 (8 IFN, 1aza)	0.55
SID-NTZ			
Age at SID-NTZ starting	37.8 (10)	41 (8.2)	0.34
EDSS at SID-NTZ starting *	1,5 (1-4.5)	2,5 (0-6)	0.41
Number on infusions *	60 (24-96)	60 (36-72)	0.81
ARR*	0 (0-0.50)	0 (0-0.20)	0.42
EID-NTZ			
Age at EID-NTZ starting	42.4 (9.6)	45.6 (8.2)	0.30
EDSS at EID-NTZ starting*	1.75 (1-4.5)	2.5 (0-6)	0.24
Number on infusions*	42 (18-42)	42 (24-42)	0.74
ARR*	0 (0-0.3)	0 (0-0.3)	0.71
Radiological activity*	0 (0-0.28)	0 (0-0.25)	0.82
JCV Index *	3.54 (1.29-4.42)	0.46 (0-1.41)	**<0.0001**

*Quantitative variables expressed as medians followed by their interquartile range in parenthesis.

ARR, Annualized relapse rate; Aza, azathioprine; DMTs, disease modifying therapies; EID-NTZ, natalizumab in extended interval dosing; IFN, interferon; SID-NTZ, natalizumab in standard interval dosing.

It has been highlighted in bold as differences between groups were significant.

### Course of switchers from natalizumab in EID to ocrelizumab

In all the switchers the reason for discontinuing EID-natalizumab was to minimize the PML risk. Patients who switched to ocrelizumab had significantly higher JCV titers in comparison to those patients who continued treatment with natalizumab (3.54 vs 0.46; p<0.0001). The washout period varied between 6 and 8 weeks (6 weeks in 12 patients, 7 weeks in 1 patient, and 8 weeks in 4 patients). All patients received intravenous methylprednisolone (1 g monthly for 3 months) during switching. After the washout period, all subjects were treated with the scheduled 2 300 mg infusions on days 1 and 15, followed by 600 mg biannual infusions. During the washout period, none of the patients experienced clinical or radiological reactivation of the disease.

**Figure 1:**
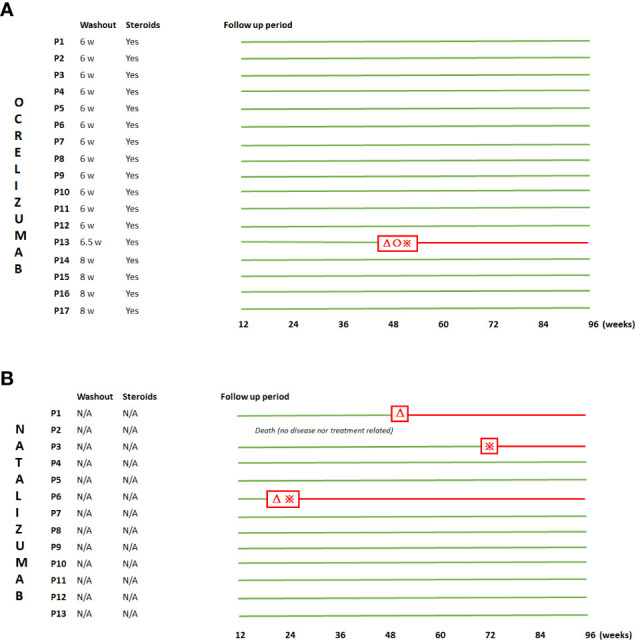
Graphic demonstration of the disease course during the whole follow-up period. Both ocrelizumab-switchers **(A)** (P1-P17) and EID-natalizumab stayers **(B)** (P1-13) are represented. Among the former, washout periods as well as the empiric steroids administration are shown. Red triangles, red crosses, and red circles, refer to clinical relapse, radiological activity and disability progression, respectively. Patients maintaining a NEDA-3 status are represented in green lines.

During the 96-week follow-up period, only one patient experienced a clinical relapse in week 48 post switch. Apart from this, no other patients previously treated with natalizumab in EID showed radiological activity or disability progression (**Figure 1**).

**Figure 2:**
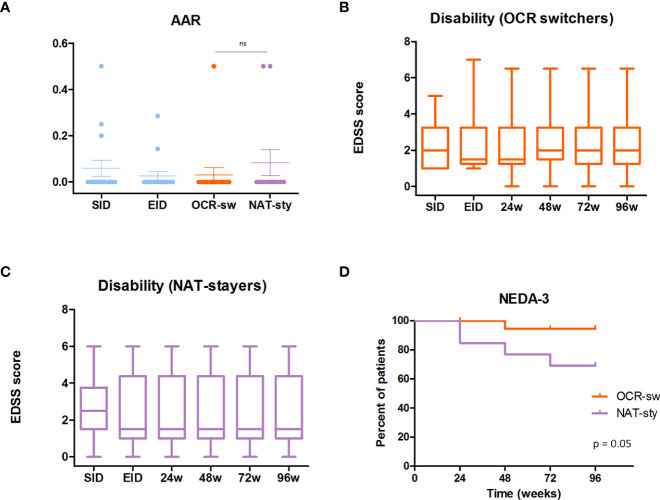
Ocrelizumab-switchers vs EID-natalizumab stayers. **(A)**. Annualized relapse rate (AAR) in ocrelizumab-switchers (orange) and in extended interval dosing (EID)-natalizumab stayers (purple). Previous AAR while treated with natalizumab in standard interval dosing (SID) and in EID (light blue). **(B)**. Disability progression in ocrelizumab-switchers at 24,48 72 and, 96 weeks. **(C)**. Disability progression in EID natalizumab stayers at 24,48 72 and, 96 weeks. **(D)** 96-week comparison of NEDA-3 status beteen ocrelizumab switchers (orange) and EID-natalizumab stayers.

Globally, ARR and radiological activity were 0.03 and 0.03 respectively. No significant ARR differences were found when comparing the ocrelizumab treatment period with natalizumab in SID and natalizumab in EID (p = 0.59) (**Figure 2A**). Regarding disability, assessed by the EDSS score, it remained stable during the whole follow-up period (p = 0.94) (**Figure 2B**).

Of note, no serious adverse events appeared in any patient treated with ocrelizumab during the 96 weeks-follow up period.

### Course of stayers in natalizumab in EID and group comparison

During the 96-week follow-up, 1 patient who stayed on natalizumab in EID died in week 4 due to an unrelated event. Two of the remaining patients showed clinical and/or radiological activity at weeks 12 and 48 and 12 and 60, respectively (**Figure 1**).

Regarding the inflammatory activity, no significant differences were detected between switchers to ocrelizumab and EID-natalizumab stayers in terms of ARR (0.03 vs 0.08, p= 0.41) (**Figure 2A**) and radiological activity (0.03 vs 0.08, p = 0.41). At the end of the follow-up period there were no differences in the EDSS in switchers as compared to non-switchers (median/mean 2/2.52 vs 1.5/2.41; p=0.63)(**Figures 2B, C**)

Respecting global control of the disease, assessed by the proportion of patients maintaining a NEDA-3 status in week 96, it was slightly superior in those subjects treated with ocrelizumab than in those staying with natalizumab (94% vs 69%, p = 0.05) (**Figure 2D**).

## Discussion

Natalizumab is one of the most effective drugs for the treatment of MS, controlling the inflammatory activity and preventing the disability progression. Yet, its use is commonly restricted due to an increased risk of PML (5, 21). Different strategies have been postulated to minimize the PML risk in patients receiving natalizumab, including the extension of the dose interval to 6-8 weeks or the switch to another high efficacy therapy. EID regimens have been proved to be an effective alternative with a significant reduction in the PML risk (6), but not completely excluding the possibility of this complication (22). On the other hand, switching to another highly effective therapeutic agent has been associated to an increased risk of disease reactivation (23). In line with other studies and the NOVA trial, our cohort of patients treated with natalizumab in EID following natalizumab in SID exhibited a favourable control of the disease (6, 10, 24). In our series, the administration of natalizumab in EID following prior natalizumab in SID successfully preserved both clinical and radiological activity and prevented disability progression in MS. There is not a strict definition of EID but in the vast majority of reported studies it ranged from 6 to 8 weeks. In the phase III NOVA trial, natalizumab administration was extended to 6 weeks (6). Differently, in the present study, patients were treated with natalizumab every 8 weeks during the extension period. Although it could be speculated that larger administration intervals might decrease the drug effectiveness (25), in a previous study involving this patient population, we did not find significant differences between SID administration and the 8-week extension (12).

Concerning switching strategies in patients at high PML risk, several studies have been published in patients treated with natalizumab in SID but only one in EID (19). The decision of switching to ocrelizumab was based on several facts, including the high effectiveness of the drug, its rapid therapeutic effect, and the favourable safety profile of the anti-CD20 agents. In keeping with this, we did not find significant adverse reactions among ocrelizumab switchers. Regarding the PML risk, recent reports suggested that some treatments for MS could modify JCV index values (24). In this sense, therapies with a T- and/or B -depleting mechanism of action were associated with a significant reduction in the JCV index, suggesting new possible sequencing strategies potentially maximizing disease control while reducing the PML risk (24).

In the absence of an alternative therapy, natalizumab cessation is associated with disease reactivation, with both radiological and clinical relapses gradually increasing 3-4 months after natalizumab discontinuation (26, 27). On this basis, the early initiation of the new treatment might be one of the most relevant factors for reducing disease reactivation. In comparison to the previously reported study (17), the washout period was slightly shortened in our series. As an adjuvant therapy, we administered monthly intravenous steroids during the first 12 weeks. This therapeutic strategy has been previously used, particularly in patients under natalizumab in SID (28). None of our patients experienced either clinical or radiological activity during first 24 weeks after natalizumab discontinuation. Thus, combining our data with the data published by Mancinelly et al, only 7 out 59 patients showed disease reactivation after switching from natalizumab in EID to ocrelizumab (19).

With respect to the long-term effects of ocrelizumab administration after natalizumab in EID, our series has the longest follow-up among published series. In this cohort of patients, ocrelizumab following natalizumab in EID was an effective agent to prevent clinical and radiological activity, as well as disability progression. Remarkably, our series of ocrelizumab switchers coming from natalizumab in EID showed a better control of the disease than those previously reported in the literature that switched from natalizumab in SID to ocrelizumab (14, 16).

The better control of the disease with ocrelizumab following EID in comparison to SID regimens may be related to the fact that patients treated with extended dosing might have less aggressive/inflammatory forms of MS.

In our study, no significant differences in ARR, radiological activity or disability progression were found between the subcohorts of patients switched to ocrelizumab and those continuing natalizumab in EID. Nevertheless, combined analysis of these parameters by the NEDA-3 index suggested a somewhat more favourable profile of ocrelizumab.

This study entails some limitations, including its observational design, the retrospective collection of clinical data, the small sample size and the reduced number of study outcomes, lacking, for instance, quality of life measurements. Thus, the results should be interpreted cautiously, and the conclusions considered as preliminary, pending further trials with larger number of patients. Ideally, those studies should include other quality of life and cognitive outcomes, thus helping clinicians to better recognize subtle degrees of disease progression. By contrast, the systematic neuroimage evaluation, as well as the duration of the follow-up, the largest published so far to our knowledge, are the main strengths.

In conclusion, these data suggest that ocrelizumab administration following natalizumab in EID is an effective and safe strategy in patients at high risk of PML. Remarkably, shortening the washout period together with the administration of intravenous steroids during the first 12 weeks may help to reduce the risk of disease reactivation. Altogether, these results suggest that switching from natalizumab in EID to ocrelizumab can be considered as a possible therapeutic choice to mitigate both the PML risk and the disease reactivation in MS patients at high risk of PML.

## Data availability statement

The original contributions presented in the study are included in the article/supplementary material. Further inquiries can be directed to the corresponding author.

## Ethics statement

The studies involving human participants were reviewed and approved by CEIm Cantabria. Written informed consent for participation was not required for this study in accordance with the national legislation and the institutional requirements.

## Author contributions

PS-S and CB-R: data collection, analysis, writing. PH-M: data collection, analysis. ME-M: data collection, revision. MT-B and CM-G: radiological analysis. FU: data collection, revision. YJ-L, MM, JRS, SS, and MD-A: data collection, critical revision. JR: conception, analysis, final revision. All authors contributed to the article and approved the submitted version.

## References

[1] Stuve O Bennett JL . Pharmacological properties, toxicology and scientific rationale for the use of natalizumab (Tysabri) in inflammatory diseases. CNS Drug Rev (2007) 13(1):79–95. doi: 10.1111/j.1527-3458.2007.00003.x 17461891 PMC6494150

[2] Kleinschmidt-DeMasters BK Tyler KL . Progressive multifocal leukoencephalopathy complicating treatment with natalizumab and interferon beta-1a for multiple sclerosis. N Engl J Med (2005) 353(4):369–74. doi: 10.1056/NEJMoa051782 15947079

[3] Tan CS Koralnik IJ . Progressive multifocal leukoencephalopathy and other disorders caused by JC virus: clinical features and pathogenesis. Lancet Neurol (2010) 9(4):425–37. doi: 10.1016/S1474-4422(10)70040-5 PMC2880524 20298966

[4] Chisari CG Comi G Filippi M Paolicelli D Iaffaldano P Zaffaroni M . PML risk is the main factor driving the choice of discontinuing natalizumab in a large multiple sclerosis population: results from an Italian multicenter retrospective study. J Neurol (2022) 269(2):933–44. doi: 10.1007/s00415-021-10676-6 34181077

[5] Kappos L Bates D Edan G Eraksoy M Garcia-Merino A Grigoriadis N . Natalizumab treatment for multiple sclerosis: updated recommendations for patient selection and monitoring. Lancet Neurol (2011) 10(8):745–58. doi: 10.1016/S1474-4422(11)70149-1 21777829

[6] Foley JF Defer G Ryerson LZ Cohen JA Arnold DL Butzkueven H . Comparison of switching to 6-week dosing of natalizumab versus continuing with 4-week dosing in patients with relapsing-remitting multiple sclerosis (NOVA): a randomised, controlled, open-label, phase 3b trial. Lancet Neurol (2022) 21(7):608–19. doi: 10.1016/S1474-4422(22)00143-0 35483387

[7] Bomprezzi R Pawate S . Extended interval dosing of natalizumab: a two-center, 7-year experience. Ther Adv Neurol Disord (2014) 7(5):227–31. doi: 10.1177/1756285614540224 PMC4206618 25342976

[8] Yamout BI Sahraian MA Ayoubi NE Tamim H Nicolas J Khoury SJ . Efficacy and safety of natalizumab extended interval dosing. Mult Scler Relat Disord (2018) 24:113–6. doi: 10.1016/j.msard.2018.06.015 29982107

[9] Clerico M De Mercanti SF Signori A Iudicello M Cordioli C Signoriello E . Extending the interval of natalizumab dosing: Is efficacy preserved? Neurotherapeutics (2020) 17(1):200–7. doi: 10.1007/s13311-019-00776-7 PMC7007494 31452081

[10] Zhovtis RL Frohman TC Foley J Kister I Weinstock-Guttman B Tornatore C . Extended interval dosing of natalizumab in multiple sclerosis. J Neurol Neurosurg Psychiatry (2016) 87(8):885–9. doi: 10.1136/jnnp-2015-312940 26917698

[11] Chisari CG Grimaldi LM Salemi G Ragonese P Iaffaldano P Bonavita S . Clinical effectiveness of different natalizumab interval dosing schedules in a large Italian population of patients with multiple sclerosis. J Neurol Neurosurg Psychiatry (2020) 91(12):1297–303. doi: 10.1136/jnnp-2020-323472 33055141

[12] Riancho J Setien S Sanchez de la Torre JR Torres-Barquin M Misiego M Perez JL . Does extended interval dosing natalizumab preserve effectiveness in multiple sclerosis? a 7 year-retrospective observational study. Front Immunol (2021) 12:614715. doi: 10.3389/fimmu.2021.614715 33841397 PMC8027344

[13] Bigaut K Kremer L Fabacher T Ahle G Goudot M Fleury M . Ocrelizumab versus fingolimod after natalizumab cessation in multiple sclerosis: an observational study. J Neurol (2022) 269(6):3295–300. doi: 10.1007/s00415-021-10950-7 PMC8725429 34982200

[14] van Lierop Z Toorop AA Coerver E Willemse E Strijbis E Kalkers NF . Ocrelizumab after natalizumab in JC-virus positive relapsing remitting multiple sclerosis patients. Mult Scler J Exp Transl Clin (2021) 7(2):20552173211013831. doi: 10.1177/20552173211013831 34123391 PMC8175839

[15] Zanghi A Gallo A Avolio C Capuano R Lucchini M Petracca M . Exit strategies in natalizumab-treated RRMS at high risk of progressive multifocal leukoencephalopathy: a multicentre comparison study. Neurotherapeutics (2021) 18(2):1166–74. doi: 10.1007/s13311-021-01037-2 PMC8423885 33844155

[16] Levin SN Ezuma C Levine L Vargas WS Farber RS De Jager PL . Switching from natalizumab to ocrelizumab in patients with multiple sclerosis. Mult Scler (2020) 26(14):1964–5. doi: 10.1177/1352458520927631 32552363

[17] Signoriello E Lus G Bonavita S Lanzillo R Sacca F Landi D . Switch from sequestering to anti-CD20 depleting treatment: disease activity outcomes during wash-out and in the first 6 months of ocrelizumab therapy. Mult Scler (2022) 28(1):93–101. doi: 10.1177/13524585211005657 33855897

[18] Lin M Zhang J Zhang Y Luo J Shi S . Ocrelizumab for multiple sclerosis. Cochrane Database Syst Rev (2022) 5:CD013247. doi: 10.1002/14651858.CD013247.pub2 35583174 PMC9115862

[19] Mancinelli CR Scarpazza C Cordioli C De Rossi N Rasia S Turrini MV . Switching to ocrelizumab in RRMS patients at risk of PML previously treated with extended interval dosing of natalizumab. Mult Scler (2021) 27(5):790–4. doi: 10.1177/1352458520946017 32749910

[20] Thompson AJ Banwell BL Barkhof F Carroll WM Coetzee T Comi G . Diagnosis of multiple sclerosis: 2017 revisions of the McDonald criteria. Lancet Neurol (2018) 17(2):162–73. doi: 10.1016/S1474-4422(17)30470-2 29275977

[21] Polman CH O'Connor PW Havrdova E Hutchinson M Kappos L Miller DH . A randomized, placebo-controlled trial of natalizumab for relapsing multiple sclerosis. N Engl J Med (2006) 354(9):899–910. doi: 10.1056/NEJMoa044397 16510744

[22] Scarpazza C De Rossi N Tabiadon G Turrini MV Gerevini S Capra R . Four cases of natalizumab-related PML: a less severe course in extended interval dosing? Neurol Sci (2019) 40(10):2119–24. doi: 10.1007/s10072-019-03959-4 31175467

[23] Roos I Malpas C Leray E Casey R Horakova D Havrdova EK . Disease reactivation after cessation of disease-modifying therapy in patients with relapsing-remitting multiple sclerosis. Neurology (2022) 99:1926–44. doi: 10.1136/bmjno-2021-ANZAN.8 PMC9620810 35977837

[24] Ryerson LZ Foley J Chang I Kister I Cutter G Metzger RR . Risk of natalizumab-associated PML in patients with MS is reduced with extended interval dosing. Neurology (2019) 93(15):e1452–62. doi: 10.1212/WNL.0000000000008243 PMC7010325 31515290

[25] Morrow SA Clift F Devonshire V Lapointe E Schneider R Stefanelli M . Use of natalizumab in persons with multiple sclerosis: 2022 update. Mult Scler Relat Disord (2022) 65:103995. doi: 10.1016/j.msard.2022.103995 35810718

[26] Prosperini L Kinkel RP Miravalle AA Iaffaldano P Fantaccini S . Post-natalizumab disease reactivation in multiple sclerosis: systematic review and meta-analysis. Ther Adv Neurol Disord (2019) 12:1756286419837809. doi: 10.1177/1756286419837809 30956686 PMC6444403

[27] Plavina T Muralidharan KK Kuesters G Mikol D Evans K Subramanyam M . Reversibility of the effects of natalizumab on peripheral immune cell dynamics in MS patients. Neurology (2020) 95(14):661. doi: 10.1212/WNL.0000000000004485 32938789 PMC8105950

[28] Fuentes-Rumi L Hernandez-Clares R Carreon-Guarnizo E Valero-Lopez G Iniesta-Martinez F Cabrera-Maqueda JM . Prevention of rebound effect after natalizumab withdrawal in multiple sclerosis. study of two high-dose methylprednisolone schedules. Mult Scler Relat Disord (2020) 44:102311. doi: 10.1016/j.msard.2020.102311 32593958

